# The urgency of suicide prevention in Afghanistan: challenges and recommendations

**DOI:** 10.1016/j.lansea.2022.100082

**Published:** 2022-10-06

**Authors:** Zoaib Habib Tharwani, Mohammad Yasir Essar, Ramadan Abdelmoez Farahat, Jaffer Shah

**Affiliations:** a Faculty of Medicine, Dow University of Health Sciences, Karachi, Pakistan; b Kabul University of Medical Sciences, Kabul, Afghanistan; c Faculty of Medicine, Kafrelsheikh University, Kafrelsheikh, Egypt; d Global Research Group (GRG), Kafrelsheikh, Egypt; e Medical Research Center, Kateb University, Kabul, Afghanistan

According to World Health Organization (WHO), suicides are globally almost twice as more common among males than in females.^1^ However, in Afghanistan around 80% of the total suicide attempts (*n =*3000) are done by women.^2^ The same fact is supported by an official strategy report by the government of Afghanistan (GoA) which shows that majority of the victims of self-immolation were females (95%) between the ages of 14–19.^3^ The most important factors for this disparity are the gender-based violence (GBV) against women, forced marriages, lack of awareness of women's rights, the impact of the war, the custom practices such as marriage portion (Tuyana) or (bride price), and family violence.^2^, ^3^, ^4^ The most common methods of suicide in Afghanistan are self-immolation, hanging, drug overdose, and consuming chemicals such as detergent.^5^ Based on the numbers given by United Nations Population Fund, around 87% of women have been a victim of at least one form of sexual, physical, or psychological violence, and 62% have faced multiple forms of abuse.^4^

After the Taliban took over in 2021, situations became even worse, as women are currently not allowed to work or receive an education due to the restrictions and cultural norms which adds up to the suffering and leads to a rise in depression among Afghan women.^2^^,^^6^ Even though the Taliban have promised to allow women to get an education and work freely, there have been attacks reported against those who do so.^2^^,^^6^ Due to certain traditional beliefs, social stigmas, and religious misconceptions, women in Afghanistan are prevented from receiving assistance for mental health, which eliminates their only chance of help, thus leading to a higher female suicide rate.^2^^,^^3^ For the same reasons, a lot of families do not report any suicide cases or attempts in their families, either by men or women, which means that not only there is underreporting of the cases but the gender disparity in Afghanistan's suicide rates is also questionable.^2^^,^^3^ Apart from being unable to receive medical attention, if an Afghan woman manages to file a report against violence to seek justice, she is pressurized and threatened by relatives to withdraw the case before the investigation even starts.^7^

According to the data given by the World Bank, in 2019, the suicide rate in Afghanistan was reported to be 4.1 per 100,000 population and specifically the female suicide rate was 3.6 per 100,000 population.^8^^,^^9^ These rates have either remained the same or increased, as compared to the rates in 2017. Considering the factors mentioned previously, it is safe to assume that underreporting of suicide cases is a significant limitation of these rates, and that the actual number of suicide cases is probably much higher in reality. This shows the need of further research in this field in order to counter the issues mentioned above as soon as possible, to discover and reduce the suicide rates in Afghanistan.

Various actions are necessary to be implemented in Afghanistan, starting with better suicide surveillance programs to determine the specific causes of suicide and regions where special attention is required. Better surveillance can also help uncover the actual suicide rates among men which are suppressed due to Islamic beliefs.^3^ Moreover, community-based mental health programs and online psychosocial counseling, as initiated by the government, should be promoted and established in all parts of Afghanistan to provide better access to those in need.^3^ Furthermore, online teaching services should be established for women, especially in the rural areas who are unable to go to school, so that they can receive the education they deserve.^10^ In addition, the local NGOs and non-governmental sectors should work with the Taliban to improve living conditions, promote gender balance, introduce human rights promotion, and enhance public awareness regarding the prevention of suicide.^3^ As forced early marriages are a major factor affecting women in Afghanistan, strict policies should be established by the policymakers and implemented by the authorities to ensure freedom of choice and provide Afghan women the rights they deserve. These steps will help educate and eradicate the traditional stigma against mental health attention among the majority of the population and provide awareness to Afghan women about their rights.

As healthcare professionals, especially psychiatrists and psychologists, play a significant role in fighting against mental health issues, the government should build more mental health clinics in multiple areas and hire healthcare professionals to help counter the mental health problems in different parts of the country. Moreover, all healthcare workers must receive training to diagnose post-traumatic stress disorder (PTSD), which is the leading cause of depression in women, and other common mental health disorders in their patients so these cases can be reported and referred to professional psychologists.^3^^,^^4^

One of the factors that have directly been linked to higher suicide rates in Afghanistan is the accessibility to drugs.^4^ Thus, it is imperative to counter this issue by following proper protocols for dispensing these drugs and officially recording their sales to keep track. Moreover, new rehabilitation centers should be developed to reduce suicides caused by overdosing.

Lastly, another factor contributing to the high suicide rates in Afghanistan is suicide bombings, which affects not only the person committing it but also the masses in their vicinity, which is their main goal.^11^ To end suicide bombing, the first step should be identifying the factors influencing these attacks. Most cases are reported to be linked with political motives, grievances, and religious misconceptions.^11^ To address these, it is imperative to involve the intelligence agencies to intervene with such groups and involve the religious clerics to eradicate religious misconceptions by issuing verdicts forbidding these acts, targeting the root cause of such attacks.^12^

## Contributors

MYE designed the draft. ZHT, MYE wrote the first draft. RAF, JS, and MYE reviewed the literature and edited the manuscript. All authors read and approved the final manuscript.

## Data sharing statement

All data are included in the above manuscript.

## Declaration of interests

Authors declare no conflicts of interest.
